# Dementia Risk and Social Determinants of Health Among Adults Racialized as Black: A Community-Based System Dynamics Perspective

**DOI:** 10.1007/s40615-024-02242-3

**Published:** 2024-11-29

**Authors:** Jean-Francois Trani, Robbie Hart, Alexis I. B. Walker, Meena Safi, Ramkrishna K. Singh, Yiqi Zhu, Ganesh M. Babulal

**Affiliations:** 1 Brown School of Social Work, Washington University in St. Louis, St. Louis, MO, USA; 2 Department of Psychology, University of Johannesburg, Johannesburg, South Africa; 3 Institute of Public Health, Washington University School of Medicine, St. Louis, MO, USA; 4 National Conservatory of Arts and Crafts, Paris, France; 5 William L. Brown Center, Missouri Botanical Garden, St. Louis, MO, USA; 6 School of Social Work, Adelphi University, Garden City, NY, USA; 7 Department of Neurology, Washington University School of Medicine, St. Louis, MO, USA

**Keywords:** Dementia, Racial disparities, Social and structural determinants of health, Structural racism, Community-Based System Dynamics

## Abstract

**Background:**

The aging population in the USA is projected to increase significantly, with a corresponding rise in dementia cases, particularly among racial minorities. This study examines the key drivers of racial disparities in dementia risk among older Black adults in the St. Louis area, a region characterized by entrenched structural racism. Utilizing a Community-Based System Dynamics (CBSD) approach, we engaged cognitively normal Black adults (age ≥ 45) to explore the complex interplay of social and structural determinants of health (S/SDOH) affecting dementia risk.

**Methods:**

Eight CBSD workshops were conducted, during which participants identified and analyzed various factors influencing dementia risk through group model-building techniques. These workshops revealed multiple reinforcing and balancing feedback loops, highlighting the intricate relationships between trauma, health literacy, social isolation, education, healthcare access, and systemic racism.

**Results:**

There were 59 participants with an average age of 64, a majority of women (88%) and college-educated (15.9 years) residing in areas with moderately severe deprivation. The resulting Causal Loop Diagrams underscored the impact of poverty, discrimination, and limited access to quality education and healthcare on dementia risk across the lifespan. Participants proposed actionable interventions, including health information campaigns, community mobilization, and improvements in public transportation and healthcare accessibility.

**Conclusion:**

This study emphasizes the necessity of addressing S/SDOH to mitigate dementia risk among Black Americans. The findings call for targeted public health initiatives and policy changes to improve socioeconomic conditions and reduce racial disparities in dementia outcomes.

## Introduction

The population of the USA, as is the case worldwide, is projected to continue to age. The number of Americans age 65 and older is anticipated to number 84 million by 2050 [1]. As a result of the growing aging population in the USA, the number of those affected by Alzheimer’s disease and related dementia (ADRD) will increase from 6.9 to 12.7 million during the same period. The risk increases with age, with 74% of ADRD cases occurring in individuals aged 75 years or older [2]. The cost of caring for older adults with ADRD is considerably high at $305 billion in 2020 but is projected to be 2.2 trillion by 2060 [3]. Direct (medical expenses) and indirect (18.4 billion h of unpaid assistance valued at $346.6 billion) costs are a primary source of stress for caregivers [4]. Racial and ethnic diversity will increase among the general and older population. By 2060, the older Black adult population is expected to increase to 13% (from 9%), the Hispanic population will increase to 21% (from 8%), and the Asian population will increase to 8% (from 4.5%), while the non-Hispanic White population (nHW) will decrease to 55% (from 77%) [5].

Black older adults are known to be at twice the risk of ADRD compared to nHW [6–9]. Recent advancements in measuring AD pathology at the molecular level using biomarkers (imaging, cerebrospinal fluid [CSF], plasma) have led to numerous studies exploring the pathophysiological basis of existing disparities. However, the evidence suggests that biological factors are insufficient to explain these disparities [10]. For example, a study of cognitively normal participants found that Black individuals had reduced CSF levels of t-tau and p-tau_181_ compared with nHW [11]. Similarly, another study comparing Black and nHW older adults with mild cognitive impairment found that plasma levels of Aβ_42_, Aβ_40_, p-tau_181_, and neurofilament light (NfL) were consistently lower in African American individuals after adjusting for demographic characteristics, educational attainment, cognition, APOE ε4 genotype, and cardiovascular factors [12]. Conversely, more recent studies found no racial difference in CSF (Aβ_42_, t-tau, p-tau_181_) or plasma (p-tau_181_, NfL) biomarkers after adjusting for covariates [13]. Most importantly, the published studies have been criticized for a small sample size of Black participants, passive recruitment of well-educated and affluent subjects, employing expensive and impractical methods, and a failure to include social risk factors [14–16].

Emerging studies [14] are redescribing existing racial disparity in ADRD outcomes as a combination of various factors, specifically structural and social determinants of health (S/SDOH) [17]. S/SDOH represents upstream environmental factors that influence health and well-being throughout life [18, 19]. Among older adults in 2019, approximately 19% of Black and 17% of Hispanic individuals lived in poverty compared to 7% of nHW [20]. Black individuals are more likely to be born in poverty and experience more discrimination compared to nHW [21]. Moreover, Black, Hispanic, and American Indian/Alaska Native individuals are less likely to have a high school education or access to health care, including primary, emergency, and preventative care, compared to nHW. They also contend with low-quality employment positions [22] and have less access to opportunities for physical activity during their adult life [23]. These disparities persist and affect the predicted increase in diversity and life expectancy.

The St. Louis community has been shaped by a history marked by events reflecting entrenched racism towards the Black community. Built near the confluence of the Missouri and Mississippi rivers, the city was the central hub for the nineteenth-century slave trade, with enslaved Black people sent by steamboats to southern cotton plantations. Following an initial St. Louis Circuit Court decision where Dred Scott and his wife, Harriet, sued John F.A. Sandford, the Supreme Court determined that the US Constitution did not extend citizenship to Black slaves. Multiple events in the twentieth century—such as the East St. Louis “Race Riots” (1917), the US Army Chemical Corps’ Operation LAC that sprayed zinc cadmium sulfide into predominantly Black North St. Louis County (mid-1950s), the construction of the St. Louis Arch (1963–1965) that displaced 19,000 Black families, and the failure and destruction of the Pruitt-Igoe housing complex (1954–1976)—have perpetuated exclusion and violence against the Black community.

Since the 1930s, St. Louis has experienced the consequences of the Home Owners’ Loan Corporation’s system of evaluating mortgage lending risk, which categorized predominantly African American neighborhoods as “hazardous” and thus “redlined” [24]. This categorization led to the exodus of middle-class, predominantly White residents to the suburbs, while predominantly Black neighborhoods in North St. Louis suffered continuous disinvestment, including the closure of Homer G. Phillips Hospital in The Ville neighborhood [25, 26]. Redlining has had lasting effects, and today, St. Louis continues to grapple with urban areas characterized by higher poverty, segregation, and income inequality [27].

Our study aims to understand key drivers of existing racial disparities in dementia risk in the greater St. Louis area, a city and county characterized by a century of entrenched structural racism and geographic racial divides. We engaged adults who self-identified as Black in examining the complex relationship among factors influencing ADRD risk using a Community-Based System Dynamics (CBSD) approach. CBSD uses group model-building techniques to explore complex problems from the perspective of individuals engaged in the issue at stake [28, 29]. We hypothesized that social and structural determinants of health (S/SDOH) are critical in explaining the gap in dementia prevalence between Black and Non-Hispanic White racial groups. In addition, we hypothesized that a medical approach alone cannot fully address public health problems, as they involve multidimensional, complex interactions driven by balancing and reinforcing feedback mechanisms [30–32]. Lastly, we hypothesize that CBSD may empower those familiar with the problem, giving them ownership in elaborating the system and identifying actions to improve it.

## Methods

### Setting

We carried out eight CBSD workshops on factors influencing dementia risk in St. Louis at Washington University School of Medicine between March 17 and 25, 2023. This study was approved by the Washington University Human Research Protection Office Institutional Review Board (HRPO 202110136), and participants provided written consent to participate.

### Study Design and Participants

Participants were enrolled in the Aging Research Characterizing Health Equity via Social Determinants (ARCHES) study, which examined key risk factors of cognitive decline and risk of dementia among Black Americans [33]. To be part of the ARCHES study, participants had to be 45 years old or older, self-identify as Black or African American, live in the greater St. Louis, Missouri metropolitan area or neighboring Illinois, and be cognitively normal based on the Montreal Cognitive Assessment [34]. Selected participants completed a baseline cognitive assessment, a S/SDOH battery using scales reflecting the various dimensions of the National Institute of Aging (NIA) Health Research Disparities Framework (i.e., access to healthcare, neighborhood cohesion, income/wealth, religiosity, stress, and life satisfaction), and a blood draw. Participants were then invited to participate in a CBSD workshop on any of the five available dates.

**Fig. 1:**
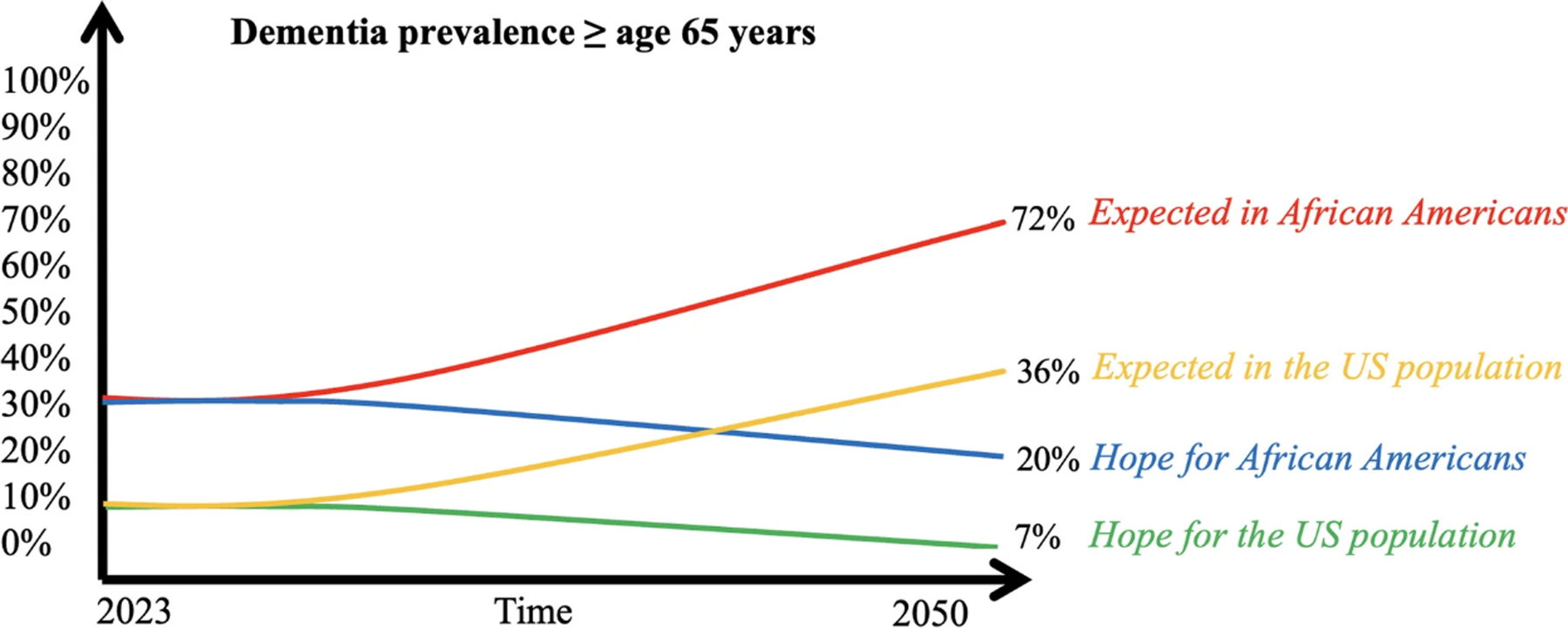
Reference model showing that Black Americans are at twice the risk of dementia

**Fig. 2:**
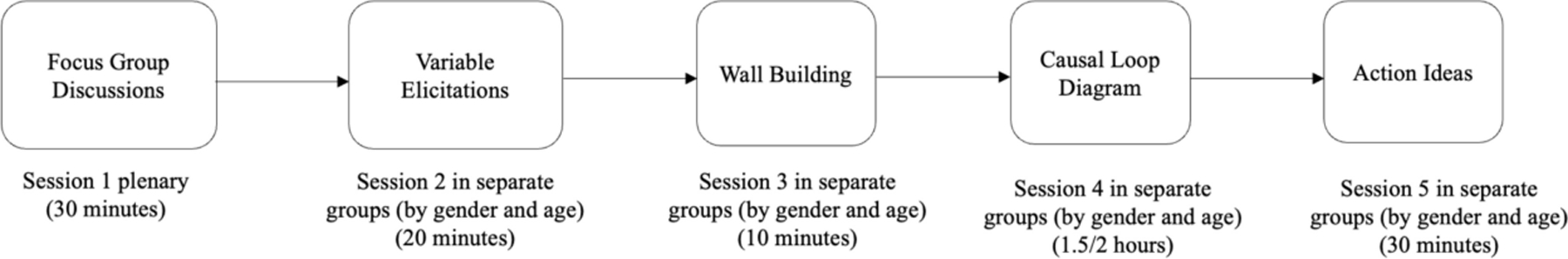
Workshop sessions

We adapted structured group exercises from *Scriptapedia*, an online open-source collection of GMB scripts, for our eight workshops [35]. Our workshops started with an introduction to the complex problem, including a reference model showing that Black Americans are at twice the risk of dementia compared to their nHW counterparts (see Fig. 1). We provided a general definition of ADRD and used “dementia,” a commonly accepted colloquialism in the community, instead of “ADRD,” which is usually used in the medical literature. The fear was that this health disparities gap would continue to widen over time if the status quo persisted. The hope was that new approaches based on an awareness of the problem might facilitate closing the racial gap by reducing the dementia risk among Black Americans. During a Focus Group Discussion (FGD), participants were encouraged to share any knowledge about the disease and how and why they believe the gap in dementia risk exists between populations racialized as Black and nHW (see Fig. 2 for the different sessions of the workshop).

Sessions three and four consisted of a variables elicitation followed by a wall-building activity first to identify the main factors influencing dementia risk of older Black St. Louisans and then organize them by themes on a wall [28]. Participants were asked to present factors/variables that they believed had the most significant impact on increasing and decreasing the risks of dementia in a round-robin manner. A facilitator with the role of “wall builder” would then paste variables shared by the participant on a wall, group them according to relevant themes, and then explain the principles they followed to organize variables. Recurrent themes were older adult physical health, older adult mental health, socioeconomic environment, family support, and healthcare system. Participants were invited to discuss the preliminary grouping of these variables and suggest modifications as they see fit.

**Fig. 3:**
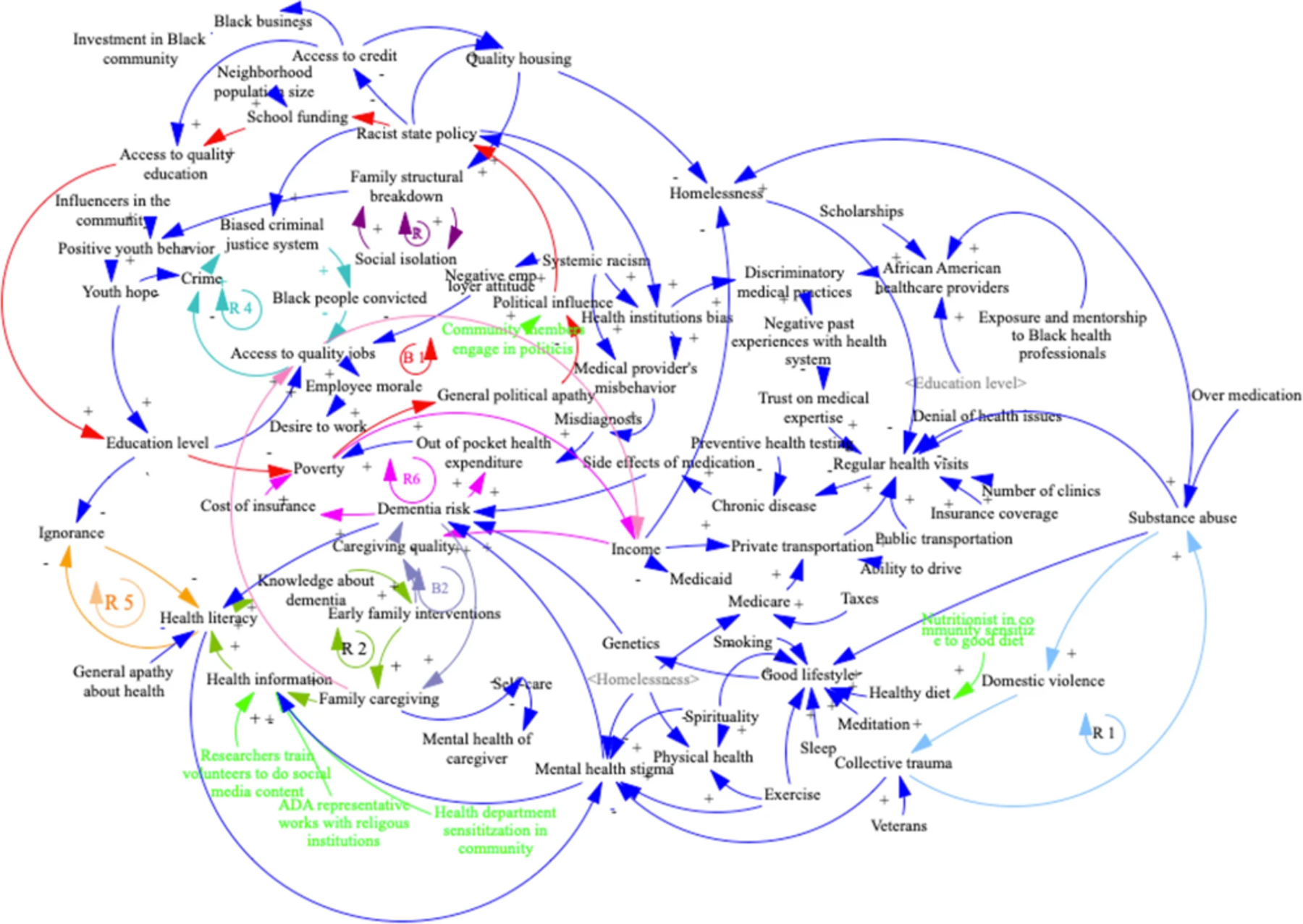
Causal Loop Diagram with six major causal feedback loops

Participants were then invited to vote silently on the essential variables they believed influence dementia risk. The fourth session engaged the participants in building a Causal Loop Diagram (CLD) followed by a final session to define action ideas that could modify the system (see Table 1 and Fig. 3). The activity was designed to explore the basic constructs and interactions between factors affecting dementia risks of older Black adults in St. Louis and to develop a common picture of the complex local dynamics and possibilities for intervention.

To ensure equal participation and inclusiveness in the workshop, a set of rules (wait for one’s turn to talk, no interruption, no side discussions, mobile phones are switched off) was discussed and collectively approved at the beginning of the workshop. Facilitators and participants collectively enforced the rules for the whole duration workshop, especially whenever dissent occurred. During the entire workshop, facilitators made a point to ensure all participants could express their views, and no voice was dominating the discussion. During FGDs and variables elicitation sessions (sessions 1 and 2), facilitators asked participants to express their views one after the other using a round-robin approach. During the CLD elaboration, facilitators were careful to give a voice to everyone, asking people who had already expressed their views to wait for others to talk. For the easiness of reading, we used the term dementia to mean either dementia severity, dementia prevalence, chronicity, and/or dementia risk as factors often influence all these aspects of the disease.

### Analysis

Initial analysis occurred throughout elaborating and refining causal connections between variables. The process was driven by the participants who collaboratively built the Causal Loop Diagrams (CLDs) through the exchange of ideas about what influences dementia. CLDs are visual diagrams (Fig. 3) built progressively through a series of participatory activities (Table 1). They are composed of three basic elements: (1) factors (or “nodes”) are variables of interest to the problem; (2) connections (or “edges”) represent causal influence between factors, and (3) causal feedback loops illustrate how a factor can influence others, often with a delay, while also being affected by those it initially influenced. These loops establish the system’s dynamic structure, adhering to straightforward principles. Arrows symbolize causal relationships (“edges”) between two factors (“nodes”). A plus sign indicates that the two factors change in the same direction: An increase in the first factor will drive an increase in the second. Conversely, a minus sign indicates that they evolve in opposite directions: An increase in the first factor will cause the second factor to decrease.

The building and review of the CLD provided the opportunity to establish face validity of the model structures of ADRD risk in St. Louis. To assess that factors introduced in the model were understandable, appropriate, and relevant to explain ADRD risk, participants were asked, based on their experiences, to suggest variables that influence ADRD risk, causal links between them, and how ADRD risk influences back factors in the system. Discussion between participants allows us to make ongoing additions to the model. Facilitators asked clarification questions for all factors and connections between factors to ensure that the wording and language were clear and understandable to all in the room. The goal was to secure a consensus between participants about its factual status. The authors documented participants’ insights through handwritten notes, photographs, and audio recordings. They used this documentation to check that the CLD was complete and accurately reflected the vision of the workshop participants. They read notes, watched pictures, and listened to audio to check each factor was correctly connected to the rest of the system and that all causal feedback loops had been identified by participants. Participants established several iterations of the model before they agreed on a shared final visual elaboration of it. All eight visual causal maps were reproduced in Vensim^®^ PLE software. Authors standardized factors based on audio recordings and notes for comparison and aggregation. This process ensured that a given concept expressed by different groups during the workshops was represented by the same factor across all CLDs.

As the eight groups were largely similar to one another in aggregate demographic characteristics, we assumed that while individual lived experiences and group dynamics gave unique insights to each CLD, all were describing aspects of the same system. Therefore, we constructed a model based on the union set of all edges identified by participants in all groups. This large model included many edges unique to a single model-building group. But some were shared among them. Focusing on these shared edges, we refined a “core” model by considering edges mentioned by at least two groups. This core model includes edges that constituted causal feedback loops (excluding exogenous nodes and incomplete causal feedback loops) and edges causally connected to the central problem of dementia risk. The fact that at least two groups identified separately a given substructure of the system adds validity to its role in shaping the dementia problem. Finally, we examined additional intervening nodes in the same cause-and-effect feedback loops to see if indirect paths increased consensus on the core model among participants.

## Results

A total of 59 Black adults participated in the 8 GMB workshops, averaging seven participants per workshop. Of those enrolled, the majority (88%) were female, the mean age was 64 years, and the average length of education was 15.8 years. Fifty-nine percent had completed a bachelor’s degree, and no one had a below-high school education (Table 2).

Models produced by each group identified an average of 71 (46–85) factors connected by an average of 115 (86–15) edges, of which an average of 9 (3–16) were action ideas. Each model represents a visual map of a complex system called a Causal Loop Diagram (CLD) with multiple causal feedback loops, as demonstrated in Fig. 3. Participants in this group identified seven major causal feedback loops. These loops comprised factors that either directly or indirectly influenced the risk, prevalence, and/or severity of dementia. Factors directly influenced dementia when the latter was embedded in the loop. The effect was indirect when one factor within the loop was independently linked to dementia, which did not belong to the causal feedback loop. The first reinforcing feedback loop (R1) in Fig. 3 had such an indirect effect on dementia: it indicated a pathway from trauma increasing substance abuse, which in turn increased domestic violence, which then influenced back trauma. From (R1), two distinct pathways influenced dementia in opposite directions. One pathway from collective trauma through reduced mental health stigma decreased dementia. Another pathway from substance abuse led to higher dementia through the negative influence of substance abuse on one’s lifestyle, increasing the risk of chronic diseases with a higher risk of side effects of medications to cure those diseases. The second reinforcing loop (R2) indicated that more health literacy translated into better knowledge about the signs of dementia. This increased the likelihood of an early family intervention. This, in turn, led to more family caregiving. This then increased the widespread dissemination of information about dementia, resulting in the spreading of health literacy. (R2) had an impact on dementia through two other feedback loops (B2) and (B3) described below.

The third reinforcing loop (R3) showed that more social isolation negatively influenced the equilibrium of the family structure. The breakdown of family structure resulted in less positive youth behaviors, reducing youth hope and increasing the likelihood of Black adults being involved in crime. This resulted in a more biased criminal justice system towards Black adults, leading to a higher number of them being convicted. With more convictions, access to quality jobs dropped for Black adults because of prejudice and legal restrictions following a conviction. Less access to quality jobs will eventually lead to more crime, increasing bias in the criminal justice system (R4). By incarcerating Black youth, our biased criminal justice system reduces their prospect of finding well-paid quality jobs, lowering their income for the duration of their active life, resulting in lower caregiving quality when the time comes, itself increasing dementia, as shown in (R7).

Reduced youth hope indirectly influences dementia risk through a pathway that starts with a lower education level: Hopeless youth do not engage in the education system. The result is a lack of education that translates into ignorance, which decreases health literacy (R5), which, through the mechanism of (R2), causes higher dementia risk. Participants also identified in (R6) that an increase in dementia risk was a source of poverty through higher healthcare out-of-pocket expenditure (R6) and higher cost of insurance (R6), which decreased family income, leading to a reduction in the quality of caregiving, which in turn increased delayed dementia risk. Furthermore, (R7) showed that more dementia risk translates into more caregiving by families, leading to lower access to quality jobs because some family members must remove their presence from the labor market to take care of older adults. This translates into lower income, reducing caregiving quality and eventually increasing dementia.

Participants also identified balancing causal loops. The first balancing loop (See B1) connected state discriminatory policies specifically targeting the Black community neighborhoods (redlining) to lower school funding. This would reduce the quality of schools and education level, which, with a delay, would increase poverty in the Black community, translating into more political apathy and less capacity to influence policymakers, leading to further discriminatory policies. The system shows that (B1) connects to dementia through poverty. Poverty is a leading cause of dementia as part of the causal feedback loop R6 and its connected loop R6 (see above). A second, more nuanced balancing loop (B2) showed that more dementia would lead to more caregiving, increasing health literacy as family members will look for more health information, resulting in better knowledge of dementia, earlier family intervention, and better caregiving quality, reducing dementia. Finally, participants in the workshop identified in (B3) that less health literacy linked to ignorance (R5) and poor education eventually fuels the stigma of mental health itself, increasing dementia by increasing distress and anxiety of those stigmatized because of their mental disease or decline.

To go beyond partial views expressed in the single CLD of Fig. 3, we calculated a model based on the union set that aggregated all eight group models. It comprised 920 edges, with 811 unique combinations of cause, effect, and polarity (whether an increase in the cause drove an increase or decrease in the effect). Sixty-three of these edges connected to action ideas decided by participants. In total, the edges connected 315 distinct factors, including 18 distinct action ideas.

**Fig. 4:**
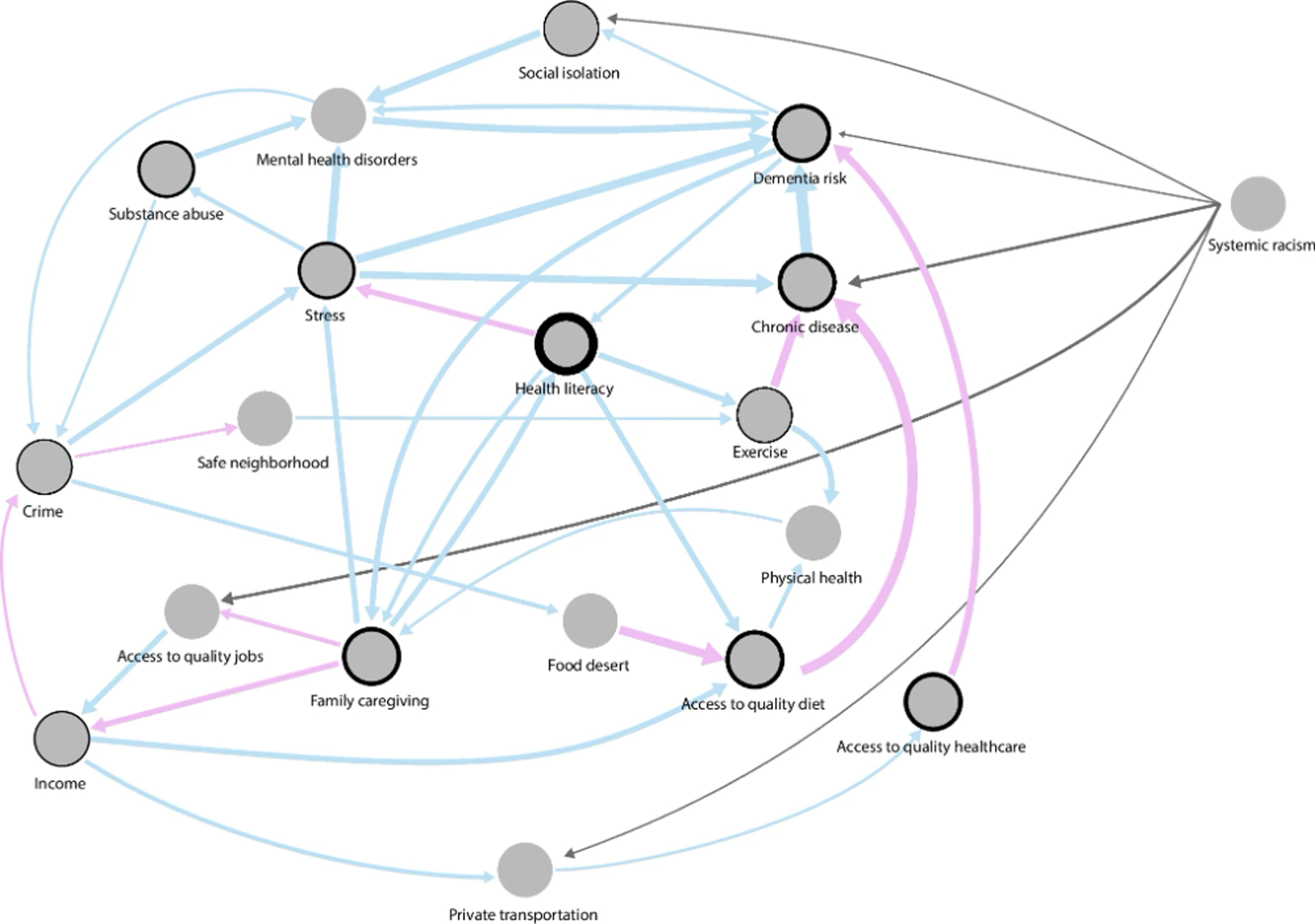
Core model across the eight groups with 18 nodes connected by 38 edges

To communicate the core attributes of this complex system (Supplementary Fig. S1), we extracted just the edges that (a) appeared in at least two group models, (b) comprised feedback loops, and (c) were connected to dementia risk. This model (Fig. 4) is simplified into a core model with shared edges indicating relative agreements between participants from the different workshops.

The core model was characterized by 18 nodes connected by 38 edges (Fig. 4). There was a consensus among participants that the risk of chronic diseases increased dementia. A large majority of participants also considered that mental health disorders increased dementia. Consensus was also found that regular physical exercise reduced the risk of chronic diseases, eventually reducing dementia. Another consensus was found around hereditary factors (genetics), where participants indicated that in some cases, dementia could be passed down from parents to their children. A causal feedback loop showed that social isolation increased mental health disorders, which in turn increased dementia, which eventually resulted in higher social isolation, creating a reinforcing causal feedback loop over time. Dementia and mental health disorders were also directly linked in a reinforcing causal loop.

Mental health disorders were influenced by a series of causal connections initiated by limited access to quality education. With limited access to education, Black Americans were more likely to have a low level of education, resulting in limited access to quality jobs, which negatively influenced their level of income. This explanation posited that higher crime resulted in higher stress in the community and higher mental health disorders that increased dementia.

Deprivation of access to quality healthcare was also identified as a cause of dementia. Numerous factors were identified around poor access, including a lack of culturally sensitive healthcare professionals, limited access to public and private means of transportation, and the latter due to lack of income. Furthermore, limited health literacy explained that more stress, less exercise, and a lower-quality diet increased the risk of chronic diseases. Other mechanisms explained the poor-quality diet of Black older adults in St. Louis. Black older adults lived in neighborhoods with more crime due to limited access to quality jobs. Crime creates insecurity, dissuades Black businesses, increases the phenomenon of food deserts that limit access to high-quality diets, and increases chronic diseases and, eventually, dementia risk.

Participants indicated that more dementia was also a cause of high out-of-pocket healthcare expenses, which increased poverty in a community already at risk due to limited income and resulted in lower-quality jobs. Higher dementia also increased family caregiving, which in turn increased poverty. More caregiving meant less time to work and earn money. Conversely, greater family caregiving translated into better health literacy, which improved exercise and a healthy diet as people became aware of its positive influence on physical and mental health and, as a result, the possibility of reducing dementia. Interestingly, more health literacy translated into less mental health stigma and better self-advocacy for access to healthcare services.

Systemic racism was identified as an exogenous factor in the system. Racism was not caused by the system itself but influenced many other factors: redlining, police response in Black neighborhoods, encounters with the criminal justice system, limited access to health professions for Black students explaining healthcare institutions bias, as well as poverty. All these were considered important life course stressors that increased dementia, as mentioned by participants in different workshops: “Racism? It affects a lot… Your self-esteem and wanting to learn more and how you handle stress… it leads to a lack of knowledge about dementia, and then you don’t know what you don’t know. […] In the African American community, we have learned to care for ourselves and not reach out to healthcare providers. With that understanding, growing up as a child, you won’t get that self-reliance.” “The stress of racism, every day you don’t know what you gonna run into… The microaggressions… like ‘your car went through’ like if it was not gonna go through, I would not have given it to you. Racism impacts you every day. From childhood, the stress is every day.” One participant summarized in one striking sentence the sentiment: “racism is the shadow which is always there.”

To improve the system, participants proposed several possible entry points for reducing dementia (Supplementary Table 2). First, they identified the need for more health information about Alzheimer’s disease and the factors influencing it. They envisioned a multipronged information campaign involving various actors and different ways to convey messages about disease prevention, symptoms, processes, and information about where to go for support. Actors included the State health department, research institutions, medical schools, and charities. Participants anticipated that there would be collaboration to establish communication material for dissemination on social media to Black charities, churches, and political organizations, as well as through community-based education programs. Second, they suggested that community leaders mobilize Black communities to vote for elected officials who expressed targeted interest in changing the status quo regarding access to healthcare by extending public transportation, outreach healthcare programs, or subsidies for mobile or community-based clinics. The suggested action ideas influenced the system in multiple ways. Participants identified 18 action ideas affecting multiple core nodes or had multiple pathways to affect the same core node (58 in total, See Supplementary Table 3).

## Discussion

Our study examined the outcomes of a series of participatory GMB workshops involving adults racialized as Black in determining factors that influence dementia. Through Community-Based System Dynamics, a participatory method that allows individuals embedded in a complex problem to develop a model of the system around this problem, older Black adults identified a series of biological but mostly modifiable S/SDOH factors involved in dementia and the relationships between them. We developed a general model of dementia in St. Louis from the standpoint of the community. In the absence of effective disease-modifying treatment and a vaccine, this study contributed to identifying modifiable S/SDOH risk factors and their interactions with dementia in the Black community. It also engaged in discourse about strategies for possible prevention and delay of onset, reifying that public health initiatives are of utmost importance [36].

Participants derived some of the evidence established in recent dementia research about causal factors and processes leading to higher dementia among Black Americans. They identified both the role of genetics, various chronic diseases, and mental health conditions—particularly depression—in increasing dementia, even mentioning multimorbidity as increasing the risk, substantiating recent findings [37–39]. More importantly, participants went beyond the pathophysiological explanation of why Black adults face higher dementia to examine how life course social exposures may influence dementia.

Participants examined the complex, dynamic, and interactive causal pathways between contextual factors that define the Black St. Louisan environment and the risk, level, and severity of dementia they face. This included poor education quality, limited healthcare access, lack of healthy food options, lower financial resources, higher unemployment, substandard housing, higher exposure to crime and pollution, and discrimination. In particular, they identified the causal link between unhealthy lifestyles characterized by the absence of physical activity, poor access to a balanced diet, the role of alcohol substance abuse, and dementia, corroborating existing scientific findings [40].

Participants also indicated that social isolation, defined as living alone, having few or no friends, and limited or no family relationships, was considerably affecting older Black adults in St. Louis. Social isolation and the resulting feeling of loneliness, defined as subjectively perceived social isolation, are underestimated major public health problems, particularly later in life [41]. Both had an impact on depression and other mental disorders and, eventually, on dementia, a relationship increasingly identified in multiple studies, including among Black Americans [42–44].

Stress was identified as a significant causal factor of chronic diseases and mental health conditions. Stress was found to result from unsafe neighborhoods due to crime, itself resulting from poverty, low education, and limited quality of employment opportunities. Participants found that systemic racism and redlining largely explained those poor S/SDOH, a source of stress among Black individuals. Coping mechanisms damaging to health, such as substance abuse (drug, alcohol) but also high-fat, calorie-dense food, have been used to deal with stress in poor communities [45, 46].

Systemic racism also explains the poor quality of healthcare received. Redlining and poverty explain barriers to healthcare access, diagnosis, treatment, and utilization [47]. Financial barriers to care, mainly because of low income and the absence of insurance, have been shown to affect more Black Americans, leading to a later and lower rate of dementia diagnosis [48]. Enduring mistrust towards healthcare professionals was explained by past experience, having to deal with providers from different racial and ethnic backgrounds that are not culturally sensitive, and perceived discrimination or racial biases, which results in poor treatment adherence [49, 50]. All these S/SDOH factors explain disparities in the burden of dementia between racial groups observed in St. Louis.

Our study is based on the combination of CBSD and network analysis, which together allow us to objectivize the subjective perspectives of individuals embedded in the complex problem of factors influencing dementia risk, prevalence, and severity. It will enable us to identify the shared structural elements of the system. This strength comes with some limitations. There were only eight workshops, including 59 older adults with limited representation of men and those with low socioeconomic and educational backgrounds. Therefore, we need to use caution in generalizing our findings to the whole county of St. Louis or other similar cities. Furthermore, we did not comprehensively address the issue of underrepresentation of racial/ethnic minorities in dementia research. Participants were predominantly from a middle-class background because of the initial difficulty in finding individuals from underprivileged backgrounds. Authors have been actively addressing this limitation through effective recruitment methods, including the use of media, speaking engagements in the community, or participation in local community events [51]. Replication through multiple workshops with older Black adults stratified by gender and socioeconomic background is ongoing to strengthen our findings and allow system components that may be associated with specific participant characteristics. Finally, it is essential to consider the role of facilitators with an academic knowledge of dementia in St. Louis. The different models stem from the collaboration between facilitators’ prompts and participants’ understandings and perspectives, explaining differences in the final maps obtained.

S/SDOH are essential factors influencing dementia risk in underprivileged urban Black neighborhoods. A significant body of literature suggests that improving S/SDOH resource allocation and public health policy and education can improve outcomes [52–55]. Many causal processes identified by study participants need further research as they remained understudied: causal pathways between social isolation, loneliness, and dementia or the effect of poverty and socioeconomic disparities on dementia are among them. Our findings call for targeted, effective, and well-resourced interventions to address those multiple dimensions of socioeconomic disadvantages. Participants suggested investment in public transportation, dementia outreach programs, accessible medical care, healthy food providers, well-resourced public schools, and better-quality jobs.

## Supplementary Material

Supplementary File2

Supplementary File1

## Figures and Tables

**Table 1:** Group model building session agenda and description of “Scripts”

Activity	Script	Description
Introduction	N/A	Introduction of group model building as a technique/tool (conceptual and ways of thinking), dementia, and of the comparative risk between Black and nHW Americans
FGD	Guidelines	What is your knowledge of dementia? Where did you learn from it? Do you think there is a similar gap in St. Louis between Black and nHW older adults?
Variable elicitation	Variable elicitation; nominal group technique	Participants responded to the prompt: “What influences dementia risk among Black older adults in St. Louis?” Each was given a stack of white A4 paper to elaborate on factors or variables influencing dementia risk. Participants were encouraged to write as many variables as come to mind, one on each sheet, and stack them for sharing in two piles, one for positive factors and one for negative factors. Facilitators used a nominal group technique to share variables. Shared variables were posted on the wall of the room in thematic groups
Stars	Dots	We used the “Dots” script elaborated in *Scriptapedia*. Each participant was given five sparkly star stickers and asked to vote for which variables from the previous variable elicitation exercise were most important
Causal Loop Diagram Elaboration	Initiating and elaborating a Causal Loop Diagram	The highest-voted variables were selected from the “Dots” exercise and were used to start the model on a wall. These variables were chosen both to reflect the priorities of participants and to model causal linkages with both positive and negative polarities. After demonstrating causal links, participants were asked to nominate variables that cause changes in the variables included on the wall. Participants were asked if they identified feedback effects. Facilitation process respected the “Initiating and Elaborating a Causal Loop Diagram” script
Model review	Model review	At the end of the CLD elaboration activity, facilitators summarized the process of building the Causal Loop Diagram and showed important feedback loops identified by participants. The facilitator then identified variables that did not have a clear cause and explained the concept of “exogenous” variables. The facilitator ended with an open discussion about the potential use of the method and model findings to suggest policy changes
Action ideas	Identify and prioritize actions	Participants suggested actions that could be taken to improve the system using sheets of paper and writing one idea per sheet. Ideas were shared one by one in a round-robin and placed by the modeler on a graph with the *x*-axis representing a continuum from easy to do until hard to do and a *y*-axis representing a continuum from low impact to high impact. Ideally, participants are looking for the sweet spot of easy-to-do and high-impact ideas. Participants discuss ideas and place them on the CLD where they could influence the system

**Table 2:** Participants’ characteristics

	Overall (*N* = 59)
Age	
Mean (SD)	64.2 (9.72)
Median [min, max]	65.0 [45.0, 92.0]
Gender	
Female	52 (88.1%)
Male	7 (11.9%)
Education (in years)	
Mean (SD)	15.8 (2.78)
Median [min, max]	16.0 [12.0, 29.0]
National area deprivation score	
Mean (SD)	70.4 (26.8)
Median [min, max]	79.0 [9.00, 100]
State area deprivation score	
Mean (SD)	6.31 (3.38)
Median [min, max]	7.00 [1.00, 10.0]

## Data Availability

Data can be made available upon request.
