# Supplementary figures and images for: Dementia Risk and Social Determinants of Health Among Adults Racialized as Black: A Community-Based System Dynamics Perspective
Source: J Racial Ethn Health Disparities. 2024 Nov 29;13(1):264–74. doi: 10.1007/s40615-024-02242-3 (PMC12119968; doi:10.1007/s40615-024-02242-3)

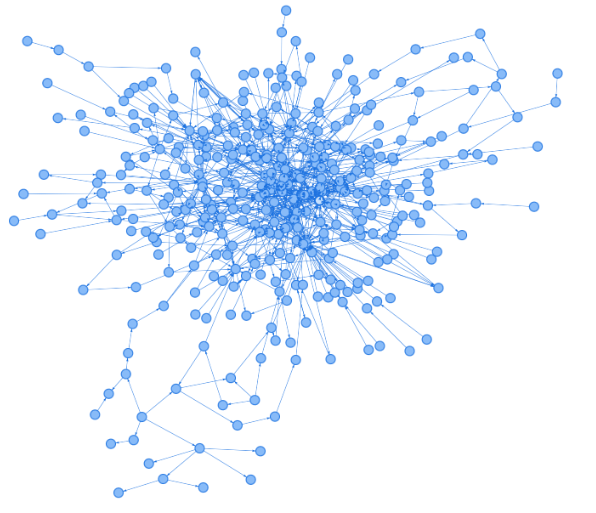

Supplement: Supplementary file 1 — Supplementary file1 (DOCX 198 KB) [file 40615_2024_2242_MOESM1_ESM.docx]
