# Supplementary material for: Dementia Risk and Social Determinants of Health Among Adults Racialized as Black: A Community-Based System Dynamics Perspective
Source: J Racial Ethn Health Disparities. 2024 Nov 29;13(1):264–74. doi: 10.1007/s40615-024-02242-3 (PMC12119968; doi:10.1007/s40615-024-02242-3)
Supplement: Supplementary file 2 — Supplementary file2 (DOCX 33 KB) [file 40615_2024_2242_MOESM2_ESM.docx]

Supplementary Table 1: Edges shared by at least two Causal Loop Diagrams

| Edges | Cause | Polarity | Effect | Total models | Models (direct links) | models (indirect links) | indirect paths |
| --- | --- | --- | --- | --- | --- | --- | --- |
| 1 | Chronic disease | increases | Dementia risk | 8 | 1,3,4,5,6 | 2, 7, 8 | 2: Chronic disease + Side effects of medication + Dementia risk, 7: Chronic disease - Physical health + Family caregiving + Private transportation + Education level + Socialization - Dementia risk, 8: Chronic disease - Brain health - Dementia risk |
| 2 | Access to quality diet | decreases | Chronic disease | 7 | 4,5,6,7,8 | 2, 3 | 2: Access to quality diet + Good lifestyle - Genetics + Dementia risk + Family caregiving - Income + Private transportation + Regular health visits - Chronic disease, 3: Access to quality diet - Obesity + Chronic disease |
| 3 | Stress | increases | Mental health disorders | 6 | 3,4,5,6,7,8 |  |  |
| 4 | Food desert | decreases | Access to quality diet | 6 | 1,4,5,6,8 | 7 | 7: Food desert - Access to quality food + Access to quality diet |
| 5 | Exercise | decreases | Chronic disease | 6 | 1,4,7,8 | 2, 5 | 2: Exercise - Mental health stigma + Dementia risk + Family caregiving - Income + Private transportation + Regular health visits - Chronic disease, 5: Exercise - Dementia risk + Mental health disorders - Access to quality diet - Chronic disease |
| 6 | Stress | increases | Dementia risk | 6 | 4,6 | 3, 5, 7, 8 | 3: Stress + Mental health disorders - Brain activity - Dementia risk, 5: Stress - Access to quality diet - Dementia risk, 7: Stress - Physical health + Family caregiving + Private transportation + Education level + Socialization - Dementia risk, 8: Stress + Mental health disorders + Dementia risk |
| 7 | Mental health disorders | increases | Dementia risk | 5 | 1,5,8 | 3, 7 | 3: Mental health disorders - Brain activity - Dementia risk, 7: Mental health disorders - Family caregiving + Private transportation + Education level + Socialization - Dementia risk |
| 8 | Stress | increases | Chronic disease | 5 | 5,6,8 | 4, 7 | 4: Stress + Dementia risk + Out of pocket cost of expenses + Caregiver stress + Chronic disease, 7: Stress - Physical health + Family caregiving + Private transportation + Education level + Access to quality jobs + Economic security + Access to quality food + Access to quality diet - Chronic disease |
| 9 | Access to quality healthcare | decreases | Dementia risk | 5 | 3,4 | 1, 6, 8 | 1: Access to quality healthcare + Access to healthcare information + Health literacy + Exercise - Chronic disease + Dementia risk, 6: Access to quality healthcare - Misdiagnosis + Stress + Dementia risk, 8: Access to quality healthcare - Chronic disease - Brain health - Dementia risk |
| 10 | Social isolation | increases | Mental health disorders | 5 | 1,5 | 3, 6, 8 | 3: Social isolation - Community network - Mental health disorders, 6: Social isolation + Depression + Mental health disorders, 8: Social isolation + Stress + Mental health disorders |
| 11 | Access to quality jobs | increases | Income | 4 | 1,2,3,4 |  |  |
| 12 | Exercise | increases | Physical health | 4 | 2,3,7 | 1 | 1: Exercise - Chronic disease + Family caregiving - Physical health |
| 13 | Health literacy | increases | Access to quality diet | 4 | 1,4,5 | 7 | 7: Health literacy - Stress - Physical health + Family caregiving + Private transportation + Education level + Access to quality jobs + Economic security + Access to quality food + Access to quality diet |
| 14 | Health literacy | increases | Exercise | 4 | 1,4,5 | 8 | 8: Health literacy - Substance abuse + Crime - Safe neighborhood + Exercise |
| 15 | Health literacy | decreases | Stress | 4 | 6,7,8 | 5 | 5: Health literacy + Exercise - Dementia risk + Mental health patient dangerous behavior + Family caregiving + Stress |
| 16 | Substance abuse | increases | Mental health disorders | 4 | 3,5,6 | 8 | 8: Substance abuse + Crime + Food desert - Access to quality diet + Physical health - Mental health disorders |
| 17 | Crime | increases | Stress | 4 | 6,7 | 5, 8 | 5: Crime + Prison population - Parental guidance - Foster care system + Stress, 8: Crime - AI WU mobile clinics + Access to quality healthcare + Health literacy - Stress |
| 18 | Dementia risk | increases | Family caregiving | 4 | 2,8 | 1, 5 | 1: Dementia risk + Health literacy + Family caregiving, 5: Dementia risk + Mental health patient dangerous behavior + Family caregiving |
| 19 | Family caregiving | increases | Health literacy | 4 | 1,8 | 2, 6 | 2: Family caregiving - Income + Private transportation + Regular health visits - Chronic disease + Side effects of medication + Dementia risk + Health literacy, 6: Family caregiving + Early retirement + Stress + Lack of interest in health knowledge + Health literacy |
| 20 | Family caregiving | decreases | Income | 4 | 2,4 | 1, 8 | 1: Family caregiving - Access to quality jobs + Income, 8: Family caregiving + Part time job - Income |
| 21 | Family caregiving | increases | Stress | 4 | 5,8 | 6, 7 | 6: Family caregiving + Early retirement + Stress, 7: Family caregiving - Caregiver leisure time - Research + Health care professional miscommunication - Equity of delivery in healthcare - Lack of reliable information - Health literacy - Stress |
| 22 | Income | increases | Access to quality diet | 4 | 6,8 | 1, 4 | 1: Income - Social isolation - Access to quality diet, 4: Income - Poverty + Food desert - Access to quality diet |
| 23 | Dementia risk | increases | Mental health disorders | 3 | 1,5,8 |  |  |
| 24 | Family caregiving | decreases | Access to quality jobs | 3 | 1,4,5 |  |  |
| 25 | Stress | increases | Substance abuse | 3 | 5,7,8 |  |  |
| 26 | Access to quality diet | increases | Physical health | 3 | 7,8 | 3 | 3: Access to quality diet - Obesity - Physical health |
| 27 | Crime | increases | Food desert | 3 | 4,8 | 5 | 5: Crime + Benefits + Food desert |
| 28 | Dementia risk | increases | Health literacy | 3 | 1,2 | 8 | 8: Dementia risk + Family caregiving + Health literacy |
| 29 | Health literacy | increases | Family caregiving | 3 | 1,4 | 7 | 7: Health literacy - Stress - Physical health + Family caregiving |
| 30 | Income | increases | Private transportation | 3 | 1,2 | 4 | 4: Income - Poverty - Private transportation |
| 31 | Crime | decreases | Safe neighborhood | 2 | 6,8 |  |  |
| 32 | Dementia risk | increases | Social isolation | 2 | 1,8 |  |  |
| 33 | Income | decreases | Crime | 2 | 6,8 |  |  |
| 34 | Mental health disorders | increases | Crime | 2 | 5,8 |  |  |
| 35 | Physical health | increases | Family caregiving | 2 | 1,7 |  |  |
| 36 | Private transportation | increases | Access to quality healthcare | 2 | 1,7 |  |  |
| 37 | Safe neighborhood | increases | Exercise | 2 | 4,8 |  |  |
| 38 | Substance abuse | increases | Crime | 2 | 5,8 |  |  |

Supplementary Table 2: Action ideas suggested by GMB workshop participants

| First core node effect | Action Idea causes |
| --- | --- |
| Access to quality diet | AI Equitable and well-funded public policies, AI Awareness about lifestyle, AI Elected officials’ sensitization |
| Access to quality healthcare | AI Access to mobile clinics, AI Research studies, AI Elected officials’ sensitization, AI Nonprofit organizations promotion of awareness of dementia, AI African American voters engaging in political life, AI Public transportation regulations |
| Chronic disease | AI Elected officials’ sensitization, AI African American voters engaging in political life, AI Community leaders engagement |
| Crime | AI Equitable and well-funded public policies, AI Access to art for youth, AI Elected officials’ sensitization, AI Nonprofit organizations promotion of awareness of dementia, AI African American voters engaging in political life |
| Dementia risk | AI Nonprofit organizations promotion of awareness of dementia, AI African American voters engaging in political life, AI Community leaders engagement, AI Awareness about lifestyle, AI Awareness about caregiving support |
| Exercise | AI Awareness about lifestyle |
| Family caregiving | AI Nonprofit organizations promotion of awareness of dementia, AI Medicare, Medicaid, and quality health insurance, AI Elected officials sensitization, AI African American voters engaging in political life |
| Health literacy | AI Elected officials sensitization, AI African American voters engaging in political life, AI Research studies, AI Nonprofit organizations promotion of awareness of dementia, AI Community leaders engagement, AI Health department and other public institutions promotion of awareness of dementia, AI Local media promotion of awareness of dementia, AI Public transportation regulations, AI African American organizations lobbying and sensitizing on dementia |
| Income | AI Elected officials’ sensitization |
| Social isolation | AI Elected officials’ sensitization, AI African American voters engaging in political life |
| Stress | AI Nonprofit organizations promotion of awareness of dementia, AI American medical association and national medical association oversight, AI Medical school training |
| Substance abuse | AI Nonprofit organizations promotion of awareness of dementia, AI American medical association and national medical association oversight, AI Medical school training, AI African American voters engaging in political life, AI Research studies, AI Community leaders engagement |

Supplementary Table 3: Action ideas with core node and pathways

| Action idea cause | First core node effect | Pathway |
| --- | --- | --- |
| AI Equitable and well-funded public policies | Access to quality diet | AI Equitable and well-funded public policies, AI Awareness about lifestyle, Access to quality diet |
| AI Awareness about lifestyle | Access to quality diet | AI Awareness about lifestyle, Access to quality diet |
| AI Elected officials’ sensitization | Access to quality diet | AI Elected officials’ sensitization, AI Equitable and well-funded public policies, AI Awareness about lifestyle, Access to quality diet |
| AI Access to mobile clinics | Access to quality healthcare | AI Access to mobile clinics, Access to quality healthcare |
| AI Research studies | Access to quality healthcare | AI Research studies, Access to quality healthcare |
| AI Elected officials’ sensitization | Access to quality healthcare | AI Elected officials’ sensitization, Access to quality healthcare |
| AI Nonprofit organizations promotion of awareness of dementia | Access to quality healthcare | AI Nonprofit organizations promotion of awareness of dementia, Police training on mental health, Criminal justice system contact, Public transportation, Access to quality healthcare |
| AI African American voters engaging in political life | Access to quality healthcare | AI African American voters engaging in political life, AI Elected officials’ sensitization, Access to quality healthcare |
| AI Public transportation regulations | Access to quality healthcare | AI Public transportation regulations, Public transportation, Access to quality healthcare |
| AI Elected officials’ sensitization | Chronic disease | AI Elected officials’ sensitization, Pharmaceutical and food corporate regulation, Appropriate medication, Chronic disease |
| AI African American voters engaging in political life | Chronic disease | AI African American voters engaging in political life, Trust on medical expertise, Poor medical treatment, Chronic disease |
| AI Community leaders engagement | Chronic disease | AI Community leaders’ engagement, AI African American voters engaging in political life, Trust on medical expertise, Poor medical treatment, Chronic disease |
| AI Elected officials’ sensitization | Chronic disease | AI Elected officials’ sensitization, Financial support for caregiving, Family caregiver quality of life, Chronic disease |
| AI African American voters engaging in political life | Chronic disease | AI African American voters engaging in political life, AI Elected officials’ sensitization, Financial support for caregiving, Family caregiver quality of life, Chronic disease |
| AI Equitable and well-funded public policies | Crime | AI Equitable and well-funded public policies, AI Access to art for youth, Youth hope and wellbeing, Crime |
| AI Access to art for youth | Crime | AI Access to art for youth, Youth hope and wellbeing, Crime |
| AI Elected officials’ sensitization | Crime | AI Elected officials sensitization, AI Equitable and well funded public policies, AI Access to art for youth, Youth hope and wellbeing, Crime |
| AI Nonprofit organizations promotion of awareness of dementia | Crime | AI Nonprofit organizations promotion of awareness of dementia, Police training on mental health, Criminal justice system contact, Prison population, Crime |
| AI African American voters engaging in political life | Crime | AI African American voters engaging in political life, AI Elected officials’ sensitization, AI Equitable and well-funded public policies, AI Access to art for youth, Youth hope and wellbeing, Crime |
| AI Nonprofit organizations promotion of awareness of dementia | Dementia risk | AI Nonprofit organizations promotion of awareness of dementia, Community network, Socialization, Dementia risk |
| AI African American voters engaging in political life | Dementia risk | AI African American voters engaging in political life, Trust on medical expertise, Poor medical treatment, Misdiagnosis, Dementia risk |
| AI Community leaders’ engagement | Dementia risk | AI Community leaders’ engagement, AI African American voters engaging in political life, Trust on medical expertise, Poor medical treatment, Misdiagnosis, Dementia risk |
| AI Awareness about lifestyle | Dementia risk | AI Awareness about lifestyle, Family financial support, Access to home health services, Changing home environment, Dementia risk |
| AI Awareness about caregiving support | Dementia risk | AI Awareness about caregiving support, Access to home health services, Changing home environment, Dementia risk |
| AI Awareness about lifestyle | Exercise | AI Awareness about lifestyle, Exercise |
| AI Nonprofit organizations promotion of awareness of dementia | Family caregiving | AI Nonprofit organizations promotion of awareness of dementia, Community network, Family caregiving |
| AI Medicare, Medicaid, and quality health insurance | Family caregiving | AI Medicare, Medicaid, and quality health insurance, Quality of nursing home, Family caregiving |
| AI Elected officials’ sensitization | Family caregiving | AI Elected officials’ sensitization, Financial support for caregiving, Family caregiver quality of life, Family caregiving |
| AI African American voters engaging in political life | Family caregiving | AI African American voters engaging in political life, AI Elected officials’ sensitization, Financial support for caregiving, Family caregiver quality of life, Family caregiving |
| AI Elected officials’ sensitization | Health literacy | AI Elected officials’ sensitization, Pharmaceutical and food corporate regulation, Health literacy |
| AI African American voters engaging in political life | Health literacy | AI African American voters engaging in political life, AI Elected officials’ sensitization, AI Health department and other public institutions promotion of awareness of dementia, Health literacy |
| AI Elected officials’ sensitization | Health literacy | AI Elected officials’ sensitization, AI Health department and other public institutions promotion of awareness of dementia, Health literacy |
| AI Research studies | Health literacy | AI Research studies, AI Nonprofit organizations promotion of awareness of dementia, Awareness about lifestyle, Health literacy |
| AI Nonprofit organizations promotion of awareness of dementia | Health literacy | AI Nonprofit organizations promotion of awareness of dementia, Awareness about lifestyle, Health literacy |
| AI Community leaders’ engagement | Health literacy | AI Community leaders engagement, AI African American voters engaging in political life, AI Elected officials sensitization, AI Health department and other public institutions promotion of awareness of dementia, Health literacy |
| AI Health department and other public institutions promotion of awareness of dementia | Health literacy | AI Health department and other public institutions promotion of awareness of dementia, Health literacy |
| AI Research studies | Health literacy | AI Research studies, Health literacy |
| AI Nonprofit organizations promotion of awareness of dementia | Health literacy | AI Nonprofit organizations promotion of awareness of dementia, Health literacy |
| AI Local media promotion of awareness of dementia | Health literacy | AI Local media promotion of awareness of dementia, Health literacy |
| AI Health department and other public institutions promotion of awareness of dementia | Health literacy | AI Health department and other public institutions promotion of awareness of dementia, AI Nonprofit organizations promotion of awareness of dementia, Health literacy |
| AI Health department and other public institutions promotion of awareness of dementia | Health literacy | AI Health department and other public institutions promotion of awareness of dementia, Dissemination of information through social networks, Health literacy |
| AI Nonprofit organizations promotion of awareness of dementia | Health literacy | AI Nonprofit organizations promotion of awareness of dementia, Access to information about health, Health literacy |
| AI Public transportation regulations | Health literacy | AI Public transportation regulations, Public transportation, Access to healthcare information, Health literacy |
| AI African American organizations lobbying and sensitizing on dementia | Health literacy | AI African American organizations lobbying and sensitizing on dementia, Health literacy |
| AI Elected officials’ sensitization | Income | AI Elected officials’ sensitization, Pharmaceutical and food corporate regulation, Price of medication, Income |
| AI Elected officials’ sensitization | Social isolation | AI Elected officials’ sensitization, Financial support for caregiving, Family caregiver quality of life, Caregiver leisure time, Social isolation |
| AI African American voters engaging in political life | Social isolation | AI African American voters engaging in political life, AI Elected officials’ sensitization, Financial support for caregiving, Family caregiver quality of life, Caregiver leisure time, Social isolation |
| AI Nonprofit organizations promotion of awareness of dementia | Stress | AI Nonprofit organizations promotion of awareness of dementia, Community network, Stress |
| AI Nonprofit organizations promotion of awareness of dementia | Stress | AI Nonprofit organizations promotion of awareness of dementia, AI American medical association and national medical association oversight, AI Medical school training, Culturally aware healthcare professionals, Primary healthcare professionals neglect, Misdiagnosis, Stress |
| AI American medical association and national medical association oversight | Stress | AI American medical association and national medical association oversight, AI Medical school training, Culturally aware healthcare professionals, Primary healthcare professionals neglect, Misdiagnosis, Stress |
| AI Medical school training | Stress | AI Medical school training, Culturally aware healthcare professionals, Primary healthcare professionals neglect, Misdiagnosis, Stress |
| AI Nonprofit organizations promotion of awareness of dementia | Substance abuse | AI Nonprofit organizations promotion of awareness of dementia, AI American medical association and national medical association oversight, AI Medical school training, Culturally aware healthcare professionals, Primary healthcare professionals neglect, Self medication, Substance abuse |
| AI American medical association and national medical association oversight | Substance abuse | AI American medical association and national medical association oversight, AI Medical school training, Culturally aware healthcare professionals, Primary healthcare professionals neglect, Self medication, Substance abuse |
| AI Medical school training | Substance abuse | AI Medical school training, Culturally aware healthcare professionals, Primary healthcare professionals neglect, Self medication, Substance abuse |
| AI African American voters engaging in political life | Substance abuse | AI African American voters engaging in political life, Trust on medical expertise, Poor medical treatment, Appropriate medication, Substance abuse |
| AI Research studies | Substance abuse | AI Research studies, AI Nonprofit organizations promotion of awareness of dementia, Awareness about lifestyle, Substance abuse |
| AI Nonprofit organizations promotion of awareness of dementia | Substance abuse | AI Nonprofit organizations promotion of awareness of dementia, Awareness about lifestyle, Substance abuse |
| AI Community leaders engagement | Substance abuse | AI Community leaders engagement, AI African American voters engaging in political life, Trust on medical expertise, Poor medical treatment, Appropriate medication, Substance abuse |
